# Workload and quality of life in Peruvian university professors

**DOI:** 10.15649/cuidarte.4031

**Published:** 2024-11-26

**Authors:** Claudia Melissa Mamani-Poma, Jeyzi Sarai Mamani-Churayra, William De Borba

**Affiliations:** 1 Universidad Peruana Unión, Lima, Peru E-mail: claudiampoma@upeu.edu.pe Universidad Peruana Unión Universidad Peruana Unión Lima Peru claudiampoma@upeu.edu.pe; 2 Universidad Peruana Unión, Lima, Peru E-mail: jeyzimamani@upeu.edu.pe Universidad Peruana Unión Universidad Peruana Unión Lima Peru jeyzimamani@upeu.edu.pe; 3 Universidad Peruana Unión, Lima, Peru E-mail: williamdeborba@upeu.edu.pe Universidad Peruana Unión Universidad Peruana Unión Lima Peru williamdeborba@upeu.edu.pe

**Keywords:** Workload, Quality of Life, Professors, Peru, Carga de Trabajo, Calidad de Vida, Docentes, Perú, Carga de Trabalho, Qualidade de Vida, Professores, Peru

## Abstract

**Introduction:**

The quality of life of professors is an important standard for their job performance.

**Objective:**

To determine the influence of workload on the quality of life of professors at a Peruvian university.

**Materials and Methods:**

This was a study with a quantitative, explanatory, cross-sectional approach applied to 326 active employees and professors at a Peruvian university during the years 2023 and 2024. Two questionnaires validated in Peru were administered. The questionnaires asked about sociodemographic data, SF-36 quality of life, and the workload scale. The data were analyzed using Studio R software, correlation analysis with Pearson R, and the explanatory model with linear regression methods.

**Results:**

It was found that, in the workload, an average of 11.56 was obtained, which shows a workload with low distribution among professors.

**Discussion:**

It was found that there is a positive and moderate correlation between workload and four dimensions of quality of life, which are physical role, bodily pain, emotional role, and social functioning; it was observed that these are significant predictors of workload.

**Conclusion:**

It is concluded that workload and quality of life influence each other. However, no relationship was found between the variables; important specific correlations were identified between workload and three dimensions of quality of life (emotional role, bodily pain, and social functioning).

## Resumen.

**Introducción:**

La calidad de vida del docente es un estándar importante para su desempeño laboral.

**Objetivo:**

Determinar la influencia entre la carga de trabajo y la calidad de vida de los docentes de una universidad peruana.

**Materiales y Métodos:**

Este fue un estudio con un enfoque cuantitativo, explicativo de corte transversal, aplicado en 326 empleados activos, docentes de una universidad peruana durante los años 2023 y 2024. Se administraron dos cuestionarios validados en Perú, con preguntas sobre sus datos sociodemográficos, calidad de vida SF-36 y la escala de carga de trabajo. Los datos fueron analizados mediante el software Studio R, los análisis de correlación con R de Pearson y el modelo explicativo con métodos de regresión lineal.

**Resultados:**

Se encontró que, en la carga de trabajo, se obtuvo una media de 11.56 lo cual muestra una carga de trabajo con distribución baja en los docentes.

**Discusión:**

Se evidenció que existe una correlación positiva y moderada entre carga de trabajo y cuatro dimensiones de la calidad de vida las cuales son: rol físico, dolor corporal, rol emocional y función social, se observó que estos son predictores significativos de la carga de trabajo.

**Conclusión:**

Se concluye que existe una influencia entre la carga de trabajo y la calidad de vida, a pesar de que no se encontró relación entre las variables se identificó correlaciones especificas importantes entre la carga de trabajo y tres dimensiones de calidad de vida (rol emocional, dolor corporal y función social).

## Resumo.

**Introdução:**

A qualidade de vida dos professores é um padrão importante para o desempenho do seu trabalho.

**Objetivo:**

Determinar a influência entre carga horária e qualidade de vida de professores de uma universidade peruana.

**Materiais e Métodos:**

Trata-se de um estudo com abordagem quantitativa, explicativa e transversal, aplicado a 326 funcionários ativos, professores de uma universidade peruana durante os anos de 2023 e 2024. Foram aplicados dois questionários validados no Peru, com perguntas sobre seus dados sociodemográficos, qualidade de vida SF-36 e escala de carga de trabalho. Os dados foram analisados através do software Studio R, análises de correlação com R de Pearson e modelo explicativo com métodos de regressão linear.

**Resultados:**

Verificou-se que, na carga horária, obteve-se uma média de 11.56, o que demonstra uma carga horária com baixa distribuição entre os professores.

**Discussão:**

Evidenciou-se que existe uma correlação positiva e moderada entre a carga de trabalho e quatro dimensões da qualidade de vida que são: função física, dor corporal, função emocional e função social, observou-se que estas são preditores significativos da carga de trabalho.

**Conclusão:**

Conclui-se que existe influência entre carga de trabalho e qualidade de vida, embora não tenha sido encontrada relação entre as variáveis, foram identificadas correlações específicas importantes entre carga de trabalho e três dimensões da qualidade de vida (papel emocional, dor corporal e função social).

## Introduction

University teaching is a tool for building knowledge and transforming thought as a permanent contribution to society. The university professor has several responsibilities: to enhance students' skills, guide their knowledge acquisition, and promote positive values and attitudes, all in an environment of respect for the environment and others^1^.

The workload that professors endure due to the methodological demands that society requires and demands, the planning and elaboration of classes, among other activities, can create an exhausting cycle for physiological and mental health^2^. The World Health Organization (WHO), together with the International Labor Organization (ILO), mentioned that excessive working hours caused 750,000 deaths from strokes and ischemic heart disease in 2021^3^.

According to Castilla^4^, 59% of professors report feeling fear for no reason and frequently feeling in a bad mood. In addition, they mention that 19.4% reported having high strain or work stress, and 17% presented strain or medium stress. The consequences are evident in physical health; 46% reported cardiopulmonary symptoms, and 45% mentioned feeling weak or tired^5^. Likewise, in Peru, it was found that 40.3% of professors present emotional fatigue, 37.7% depersonalization, and 39.6% mention low levels of professional fulfillment, thus affecting the quality of life of professors^6^.

The quality of life of professors is an essential standard for their work performance because they must meet the criteria to carry out their activities and have the necessary resources for optimal development in their area^7^. For there to be an adequate quality of life, there must be job satisfaction; this determines the well-being and commitment to professor productivity^8^.

Considering that quality of life is related to environmental conditions, and support from family, friends, and colleagues^9^, it is important that the professor be rewarded and receive public and salary recognition. In this way, the professional feels satisfied with his activities and is committed to the institution^10^.

However, reality is far from what is expected. Today, there is an excessive demand for both professors and students since students are educated to produce and contribute to society. The pressure on professors due to job competition is becoming increasingly stronger^11^. Rodriguez and Huapaya^12^^)^ revealed that the workload presents risky degrees that affect workers , which could impact the professor's quality of life. In Peru, 48.3% of professors at a public university were dissatisfied with their low salaries, non-existent appointments, and dissatisfaction with the recognition of their work^13^.

Consequently, workload is a problem that triggers a series of effects that can range from an argument at work to health problems such as anxiety, depression, muscle pain, and sleep problems, among others that affect the quality of life, in addition to high stress, insomnia, hypertension, obesity, and cardiovascular diseases^14^.

Previous studies have indicated high levels of stress and health problems among university professors. However, only some have explored the influence of workload on quality of life in the specific Peruvian context. Given the above, a study was proposed to determine the influence of workload on the quality of life of Peruvian university professors.

## Materials and Methods

### Study design and population

This was a study with a quantitative, explanatory, and cross-sectional approach.

The population consisted of 723 active employees and professors of the Universidad Peruana Unión, both Peruvian and foreign professors, between 2023 and 2024. Considering that Law No. 31964 requires university professors to meet the requirements to obtain Master's and Doctorate degrees to teach at a Peruvian university, the same applies to foreigners. According to Peruvian Law, a foreign professional who has validated his/her professional title can also work as a professor if he/ she has at least a Master's or Doctorate recognized by the Ministry of Education. It was conducted using non-probabilistic accidental sampling with 326 employees as a sample. Active professors, practice supervisors, and theory supervisors were included. The study dataset is available on Mendeley data^15^.

### Variables and data collection

The Workload Scale (ECT), created by UNIPSICO, was used to evaluate the independent variable. This battery evaluates psychosocial factors at work. Gustavo Calderón, Cesar Merino, Arturo Juárez, and María Jiménez validated it in Peru. This questionnaire has six questions and only one dimension. The questions have five-level Likert-type responses ranging from "0 = Never" to "very frequently = 4." The score is obtained from a simple sum of the item responses. The questionnaire adds up to a total score of twenty-four and does not have a cut-off point; this is due to the nature of the construct measured, the variability in the tasks or working conditions evaluated by the items of the instrument, its interpretation being flexible in using quantitative and qualitative methods for its interpretation, the interpretation can be established using the mean and standard deviation of the sample or by workload categories: 19 to 24 = (very high workload), 13 to 18 = (high workload), 7 to 12 = (moderate workload) and 1 to 6 = (low workload). The instrument has construct validity with confirmatory factor analysis. It indicates adequate models in the representation of the construct. Likewise, the reliability was obtained by internal consistency with the alpha coefficient (0.803) and the omega coefficient (0.806) ^16^.

The Quality of Life (QOL) scale was used to evaluate the dependent variable. It was created by Aaronson, Acquadro, and Apolo in 1992 as an international quality-of-life assessment project. It was validated in Peru by Fernando Salazar and Eduardo Bernabé. The SF-36 version 2 consists of 36 items organized into 8 dimensions: Physical functioning (10 items), physical role (4 items), bodily pain (2 items), general health (5 items), vitality (4 items), social functioning (2 items), emotional role (3 items) and mental health (5 items). Another unique point addresses the health transition during the past year health transition (1 item). The instrument is reliable for the 8 dimensions. The scale has a score of "0 to 100 points, with zero being the worst state and 100 being the best state." The coefficient for the SF-36 questionnaire scale was 0.82; by scales, the coefficients ranged from 0.66 for the social functioning (SF) scale to 0.92 for the physical role (PR) scale^17^.

The study mentions the covariates of age, sex, marital status, educational level, work experience, number of children, and income.

Professors were invited to participate in the study through an online survey, and the objectives and purpose of the survey were provided. Those interested professors selected the option to continue with the questionnaire, thus permitting them to participate. The questionnaire included information about the study, consent, general data, the SF-36 quality of life questionnaire, and the Workload Scale.

### Data analysis

The data were analyzed using Studio R software version 3.2.2; the variables were descriptively analyzed using absolute and relative frequency in percentages, the mean, standard deviation, asymmetry analysis, and kurtosis. The correlation analysis with Pearson's R indicates that the correlation coefficient is the statistic that allows measuring the degree ofassociation oftwo linearly related variables. Its values vary between -1 and 1. Linear regression analysis methods were used for the prediction model^18^. These methods allow studying the relationship between a dependent variable (also called response or "y") and one or more independent variables (generally represented as "x"). The intercept is the value of the dependent variable "y" when the independent variable "x" equals zero. When the intercept is different from zero, it indicates that, even when the independent variables have a null value, the dependent variable will not be zero; in other words, the predicted value of the dependent variable will be equivalent to the value of the intercept. This value is useful for understanding the base behavior of the dependent variable when the independent variable (x = 0) is present. Regarding the interpretation of beta, it is considered that the magnitude of beta's values is greater than 0.30. For interpretation of the "P" value, the regression model18 is considered significant when it is less than 0.05. To indicate that the proportion or percentage of the total variation of the dependent variable Y is explained by the independent variable X, the r2 used varies in the range of 0 < r2 < 1, or 0 to 100%. The higher the r2, the greater the reliability of the predictions and the lower the predictive errors will be; values between 0.2 and 0.4 are considered acceptable. To confirm the model, multimodality was analyzed with the variance inflation factor (VIF) with a cut-off point equal to 1 and less than 5. This suggests a moderate correlation between a given predictor variable and other predictor variables in the model but does not present multimodality.

### Ethical Aspects

This study project was evaluated by the ethics committee of the Faculty of Health Sciences of the Universidad Peruana Unión, which was accepted with approval number 2023-CE- FCS-UPeU-201. Consequently, the recommendations of the institution's ethics committee were followed. Each participant was requested to give informed consent. During this process, the study's objectives, benefits, risks, and confidentiality of the data were explained in detail. Therefore, participation was voluntary through a consent term. Data collection then began. Ultimately, the data obtained and the Excel databases were strictly stored with restricted access, which only the researchers could access. It is worth mentioning that ethical standards outlined in the Declaration of Helsinki, the Nuremberg Code, and the Belmont Report were respected in this research.

## Results

Table 1 provides a detailed overview of the sociodemographic data. Firstly, it is noteworthy that the average age of workers is around 45.2 years, with a standard deviation of 9.2. Regarding sex, the sample is almost equally divided between men and women, with 52.46% and 47.54%, respectively. Regarding marital status, 71.16% are married, and 21.47% are single. As for the number of children, 32.51% have two children, 29.44% have one child and 20.55% have no children. Regarding academic degrees, 51.22% have bachelor's degrees, and 38.65% have master's degrees. Regarding economic income, 36.80% receive from 1,501 to 2,500 soles per month, while 34.35% receive from 2,501 to 3,500 soles per month. Regarding work experience, 41.71% have 5 to 9 years, and 33.74% have 10 years or more Table 1.

**Table 1:** Sociodemographic data

Variable	n (frequency) 326	% (percentage)
Age. Mean ± Standard deviation	45.2 ± 9.2	
Sex		
Male	171	52.46
Female	155	47.54
Marital Status		
Single	70	21.47
Married	232	71.16
Divorced	14	4.29
Widow(er)	10	3.06
Number of children		
0	67	20.55
1	96	29.44
2	106	32.51
3	45	13.80
4	9	2.76
More than 4	3	0.92
Studies		
Graduate	167	51.22
Master	126	38.65
Doctorate	33	1.01
Monthly Economic Income		
247 - 391 USD	9	2.76
391 - 652 USD	120	36.80
652 - 913 USD	112	34.35
913 - 1173 USD	57	17.48
Over 1173 USD	28	8.58
Years of work experience		
Less than 1 year	1	0.30
1 to 4 years	79	24.23
5 to 9 years	136	41.71
10 years or more	110	33.74

Table 2 provides a detailed overview of the descriptive data on the characteristics of teaching work. Firstly, it is noteworthy that 66.26% are dedicated exclusively to teaching, and 33.74% are professors with other parallel tasks. Regarding the faculty where they work, 26.9% belongs to health sciences, 21.47% to business sciences, 21.16% to human sciences and education, and 23.31% to engineering and architecture. As for working hours, 42.94% work 8 hours, followed by 26.07% with 10 hours.

**Table 2:** Characteristics of teaching work

Variable	n (frequency)	% (percentage)
Current employment status		
I am dedicated exclusively to teaching	216	66.26
In parallel to teaching, I also work in another place	110	33.74
In which Faculty do you work?		
Health Sciences	88	26.99
Business Sciences	70	21.47
Human Sciences and Education	69	21.16
Engineering and Architecture	76	23.31
Theology	23	7.05
Hours worked per day		
5	4	1.22
6	22	6.74
7	15	4.60
8	140	42.94
9	37	11.34
10	85	26.07
12	23	7.05

Table 3 presents a descriptive analysis of the two study variables and their dimensions. Quality of life has a mean of 97.85 and a standard deviation of 14.93, suggesting significant variability in the data. The distribution is skewed to the left, with a negative skewness of -0.35, indicating more people with quality of life below the average than above. However, the distribution is almost symmetrical overall.

Regarding workload, the mean is 11.56 with a standard deviation of 2.95, suggesting less variability compared to quality of life. The distribution also appears skewed to the left, with a negative skewness of -0.39, and a kurtosis indicating a relatively flattened but pointed distribution.

The dimensions of quality of life reveal varied results. Physical functioning and physical role show moderate levels on average, with standard deviations indicating moderate variability between individuals. The data distribution for these dimensions is skewed to the left, with more people below the mean.

Bodily pain, general health, vitality, social functioning, emotional role, and mental health also show moderate levels on average, with similar variability between individuals. The distributions of these data vary in skewness and shape but generally show a moderate degree of asymmetry and peakedness. It highlights that mental health has a mean that suggests a positive perception on average, with a distribution slightly skewed to the right, indicating a tendency towards good mental health in the sample.

**Table 3:** Descriptive analysis of the study variables

	Mean	Standard deviation	Skewness	Kurtosis
Quality of life	97.85	14.93	-0.35	-0.01
Physical functioning	25.7	4.1	-0.81	-0.06
Physical role	12.35	3.15	0.16	-0.57
Bodily pain	5.94	1.43	-0.23	0.39
General health	13.44	2.6	0.43	1.13
Vitality	11.19	2.67	-0.14	0.33
Social functioning	5.85	1.09	-0.18	0.59
Emotional role	8.9	2.57	0.44	-0.44
Mental health	14.48	3.2	0.3	-0.49
Workload	11.56	2.95	-0.39	0.45

*Note: M = mean; SD = standard deviation*

Table 4 shows the correlation analysis between the Workload Scale and quality of life. Although no statistically significant correlation was found between workload and overall quality of life, significant relationships were observed with some specific dimensions.

First, a moderate positive correlation was identified between the Workload Scale and the bodily pain dimension (r = 0.32, p < 0.001). This result suggests that the greater the workload perceived by professors, the greater the physical pain or bodily discomfort experienced. Furthermore, workload was also positively correlated with social functioning (r = 0.33, p < 0.001), which could indicate that higher levels of work overload are associated with a greater impact on professors' social activities and interpersonal relationships. On the other hand, weak positive correlations were found between the Workload Scale and the dimensions of physical role (r = 0.14, p = 0.011) and emotional role (r = 0.14, p = 0.012). These findings suggest that a higher workload is associated with more significant interference in professors' physical and emotional activities, although less than with bodily pain and social functioning.

**Table 4:** Correlation analysis between the study variables

Variables	Workload	
	r	P
Quality of life	0.09	0.097
Physical functioning	-0.06	0.265
Physical role	0.14*	0.011
Bodily pain	0.32***	0.000
General health	-0.02	0.698
Vitality	-0.09	0.089
Social functioning	0.33***	0.000
Emotional role	0.14*	0.012
Mental health	0.10	0.067

Note: * p < .05, ** p < .01, *** p < .001; r: Spearman Rho correlation

Table 5 shows the multiple linear regression analysis performed, which allowed us to identify significant predictors of professors' workload perceptions. The resulting model, which included the dimensions of bodily pain, social functioning, and emotional role, explained 34.1% of the variance in workload.

Three of the dimensions proved to be significant predictors. First, bodily pain emerges as the strongest predictor (P = 0.57, p < 0.001), suggesting that the greater the presence of physical pain or bodily discomfort, the greater the workload perceived by professors. This finding highlights the importance of addressing factors contributing to pain and physical discomforts, such as ergonomic conditions, long working hours, or work stress. Furthermore, social functioning was also identified as a significant predictor (P = 0.61, p < 0.001), indicating that the greater the interference in social activities and interpersonal relationships, the greater the reported workload. This result highlights the importance of promoting an adequate balance between professors' work and personal life, fostering opportunities to develop support networks and social activities.

On the other hand, emotional role was also a significant predictor (P = 0.25, p = 0.017), suggesting that the greater the interference in emotional activities, the greater the workload experienced. This finding highlights the importance of providing emotional support and effective coping strategies for professors to mitigate the impact ofjob demands on their emotional well-being.

**Table 5:** Linear regression analysis of workload on quality-of-life dimensions

Predictor	p	SE	t	P	Intercept	R2	VIF
					2.22	0.341	1.51
Physical Role	0.01	0.08	0.12	0.904			
Body pain	0.57	0.12	4.88	0.000			
Social Functioning	0.61	0.15	4.05	0.000			
Emotional Role	0.25	0.10	2.40	0.017			

## Discussion

The present research found that the workload data distribution in professors is 11.56, which shows a workload with low distribution. Likewise, a study was conducted in Metropolitan Lima in which a low workload level was observed with 6.22^19^. Similarly, a study carried out on Peruvian professors showed that they perceived the workload as low, with an average of 13.87^20^. It is worth mentioning that the populations that presented a low burden level are institutions that practice a healthy lifestyle. Similarly, a study conducted at a higher education institution in Lima observed a high workload score, alleging role conflict and ineffective coping^21^. At the same time, excessive workload is indeed a factor that triggers various problems for the physical and mental health of university professors^14^.

Regarding quality of life, it was observed that it affects different areas of health, such as mental, physical, and social health. Likewise, a study conducted in Chile shows that quality of life is affected by excessive work factors^22^. Similarly, for a professor to have an adequate quality of life, there must be satisfaction in the workplace. This determines social and individual well-being and, thus, adequate productivity in their work^8^.

According to the research objective, there is a positive and moderate correlation between the Workload Scale and the four dimensions of quality of life: physical role, bodily pain, emotional role, and social functioning; three are significant predictors of workload. Similarly, a study conducted with professors from Colombia and Chile showed that the dimensions most affected in both countries were emotional and physical roles^23^. In another article, it was possible to show that the dimensions that agree with our study are the emotional and physical roles. A similar study shows that an overworked professor needs more motivation when carrying out their work, which affects student learning and the professor's health^24^. Due to curricular activities, grading of materials or group work, pain in the upper and lower limbs, headaches, and spine pain are caused. In addition, the workload affects the social activity of a professor, generating specific problems for professor in carrying out collaborative work^25^.

On the other hand, it was found that there is no correlation between the Workload Scale and the four dimensions of quality of life, which are physical functioning, general health, vitality, and mental health.

A similar study in Brazil showed that vitality had the lowest score, followed by mental health^26^. A study also shows that healthy lifestyle practices provide energy and vitality, which explains a better quality of life. In addition, physical activity promotes levels of satisfaction with life. The stimulating action of endorphins explains these effects and consequently causes a feeling of well-being and a decrease in anxiety and stress^27^.

## Conclusion

There is an influence between workload and quality of life. However, no relationship was found between the variables of identifying important specific correlations between workload and three dimensions of quality of life (emotional role, bodily pain, and social functioning), which suggests that excessive workload negatively impacts the social functioning of professors, more significant work effort is associated with an increase in physical discomfort affecting their activities and interpersonal relationships. The quality-of-life dimensions (bodily pain, social functioning, and emotional role) were significant predictors of workload; this model explained 34.1% of the variance. It is suggested that as the workload increases, the quality of life of professors decreases.
